# Regeneration Effect of a New Bio-Based Warm-Mix Rejuvenator on Performance and Micro-Morphology of Aged Asphalt

**DOI:** 10.3390/ma17092077

**Published:** 2024-04-28

**Authors:** Zhaoyi He, Le Yu, Shiyuan You, Maorong Li, Lin Kong, Dingbang Wei

**Affiliations:** 1 School of Civil Engineering, Chongqing Jiaotong University, Chongqing 400074, China; hzyzwb@cqjtu.edu.cn (Z.H.); ysyyola@163.com (S.Y.); 2 Chongqing City Construction Investment Group Co., Ltd., Chongqing 400023, China; 3 National and Local Joint Engineering Laboratory of Traffic Civil Engineering Materials, Chongqing Jiaotong University, Chongqing 400074, China; maorongleede@163.com; 4 School of Civil Engineering, Southwest Jiaotong University, Chengdu 610031, China; konglin@my.swjtu.edu.cn; 5 Gansu Transport Planning, Survey and Design Institute Co., Ltd., Lanzhou 730030, China; weidingbang@163.com

**Keywords:** bio-based warm-mix rejuvenator, aged asphalt, road performance, micro-morphology, regeneration effect

## Abstract

The use of warm-mix recycling technology can reduce the mixing temperature and the secondary aging of binders in reclaimed asphalt pavement (RAP), which is one of the effective ways to recycle high-content RAP. In this study, the penetration, softening point, ductility, and viscosity were used to characterize the conventional physical properties of aged asphalt after regenerating, while a dynamic shear rheometer (DSR), force ductility tester (FDT), and atomic force microscope (AFM) were used to evaluate the rheological performance and micro-morphology of aged asphalt incorporating a new bio-based warm-mix rejuvenator (BWR) and a commercial warm-mix rejuvenator (ZJ-WR). The regeneration mechanism of warm-mix rejuvenators on aged asphalt was analyzed by Fourier transform infrared spectroscopy (FTIR). The results show that the new bio-based warm-mix rejuvenator can restore the conventional physical properties, low-temperature performance, and micro-morphology of aged asphalt with an appropriate dosage, but it has a negative effect on high-temperature performance. In comparison with 2D area parameters, 3D roughness parameters were more accurate in evaluating the variation in micro-morphology of aged asphalt after regeneration. The FTIR analysis results indicate that both the new bio-based warm-mix rejuvenator and the commercial warm-mix rejuvenator regenerate aged asphalt by physical action, and *A_S=O_* and *A_C-H_* values are more reasonable than the *A_C=O_* value for the restoration evaluation of aged asphalt. And the new bio-based warm-mix rejuvenator has a better regeneration effect on the performance and micro-morphology of aged asphalt than the commercial warm-mix rejuvenator.

## 1. Introduction

Nearly 800 million tons of reclaimed asphalt pavement (RAP) are produced for asphalt maintenance every year in China [1]. How to minimize the construction cost and reduce the risk of environmental pollution by the utilization of high-quality and high-content RAP has been a hot topic in the field of road engineering [2]. Typical recycling technologies mainly include cold recycling technology and hot recycling technology. Cold recycling technology has the advantage of recycling asphalt pavement at ambient temperature, but the strength and durability of cold recycled materials are relatively compromised [3]. Hot recycling technology combines aged asphalt with new asphalt (or rejuvenators) at a high temperature to improve the performance of recycled asphalt, and so far, hot recycling in-plant technology is the best way to guarantee the performance of reclaimed asphalt mixtures [4,5]. However, due to the difficulty in fully recovering performance and due to the existence of secondary aging of the RAP binder under high temperatures, it remains challenging to improve the contents of RAP with the road performance of reclaimed asphalt mixtures at the same time [6,7].

Aiming at the efficient regeneration of RAP binders, researchers mainly replenish the light components lost by oxidation or volatilization and restore the colloidal structure and physicochemical properties of aged asphalt by adding rejuvenators [8,9]. Petroleum-based rejuvenators such as aromatic oil and naphthenic oil are the most commonly used rejuvenators because of their good compatibility and similar chemical composition with asphalt [10]. However, petroleum products contain harmful substances such as polycyclic aromatic hydrocarbons, which easily produce toxic gases during mixing. Meanwhile, petroleum is a non-renewable resource, and its long-term use is not in line with the concept of sustainable development. Bio-oil is an oil-like viscous material obtained by the pyrolysis of straw, livestock manure, and other biomass, which contains elements like carbon, hydrogen, oxygen, nitrogen, and sulfur. While these elements are similar to those in asphalt, the actual content of these elements between bio-oil and asphalt is significantly different, especially the content of oxygen [11,12]. Therefore, bio-oil cannot directly substitute petroleum asphalt but has the potential to serve as a rejuvenator to improve the colloidal structure of aged asphalt by supplementing light components such as saturates and aromatics. Due to the fact that some bio-oil contains amide polar groups and nitrogen-containing functional groups that can disaggregate the asphaltene structure [13], many researchers claim that the use of bio-oil is promising as environmentally friendly rejuvenators [14,15]. In recent years, it has become a trend to replace petroleum-based rejuvenators with bio-based rejuvenators. Many studies have shown that waste plant oil, waste edible oil, and soybean oil can affect the micro-properties of aged asphalt by diluting or dissolving, thereby improving the physicochemical and rheological properties of aged asphalt [16,17,18]. However, many problems still exist that cannot be simply solved only by using rejuvenators [19], such as the secondary aging of RAP binders at high temperature and the low contents of RAP in recycled asphalt mixtures. 

One commonly used approach to solve the problems mentioned above is through Warm-mix asphalt (WMA) technology, which has the advantages of reducing the mixing temperature and greenhouse gas emissions and improving construction workability [20,21]. By adding different contents of RAP into WMA, researchers have evaluated the effects of RAP content on the rutting resistance, cracking resistance, moisture resistance, and fatigue resistance of WMA mixtures, as well as the effects of warm-mix rejuvenators on the road performance and micro-morphology of WMA mixtures containing RAP [22,23,24,25]. Then, some studies considered adding additives (organic or chemical) and rejuvenators into asphalt, respectively, and compared the rheological properties of two binders and the mechanical behavior of WMA mixtures containing RAP [6,26,27]. However, the step of adding additives and rejuvenators complicates the process of construction, leading to low efficiency in asphalt mixture production. Thus, research on how to combine additives and rejuvenators for a better warm-mix rejuvenator is increasingly becoming popular. Investigations on this new type of warm-mix rejuvenator include a broad range of topics, for instance, the conventional physical properties, rheological properties, micro-morphology, and regeneration mechanisms of warm-mix recycled asphalt. However, most of the warm-mix rejuvenators considered are petroleum-based, and bio-based warm-mix rejuvenators are rarely studied [28,29,30,31,32].

The purpose of this study is to investigate the regeneration effect of aged asphalt incorporating a new bio-based warm-mix rejuvenator (BWR) which was self-developed by the research group. The penetration, softening point, ductility, viscosity, dynamic shear rheometer (DSR), force ductility tester (FDT), and atomic force microscope (AFM) were used to evaluate the regeneration effect of BWR and ZJ-WR on the conventional physical properties, rheological properties, and micro-morphology of aged asphalt. The regeneration mechanism of BWR and ZJ-WR were analyzed by Fourier transform infrared spectroscopy (FTIR).

## 2. Materials and Methods

### 2.1. Materials

#### 2.1.1. Warm-Mix Rejuvenator

**Figure 1:**
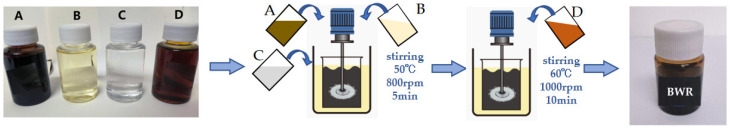
Flowchart of preparing BWR.

The bio-based warm-mix rejuvenator (BWR) is a deep brown liquid and consists of waste plant oil (A), epoxidized soybean oil (B), naphthenic rubber oil (C), and oleic acid diethanolamide (D). The mass percentages of each component in BWR are 50.1%, 9.8%, 11.2%, and 29.9%, respectively. Waste plant oil is a by-product obtained in the plant oil distillation process of fatty acids, which can supplement light components of aged asphalt. Epoxidized soybean oil is a plasticizer with good compatibility and low volatility. The polar groups in epoxidized soybean oil can interact with those in aged asphalt to improve its plasticity and thermostability [28]. Naphthenic rubber oil mainly plays the role of viscosity reduction. Oleic acid diethanolamide is a non-ionic surfactant, which has the effect of reducing the mixing temperature and improving the compatibility. As a comparison, ZJ-WR is a yellow solid-state warm-mix rejuvenator with linseed oil and glyceryl monostearate as the main components, and it is produced by Chongqing Zhongjiao Renewable Resources Development Co., Ltd. The mass percentages of linseed oil and glyceryl monostearate in ZJ-WR are 40% and 60%, respectively. The properties of BWR and ZJ-WR are listed in Table 1, and the detailed preparation procedure of BWR is described in Figure 1.

#### 2.1.2. Aged Asphalt and Its Rejuvenation

**Figure 2:**
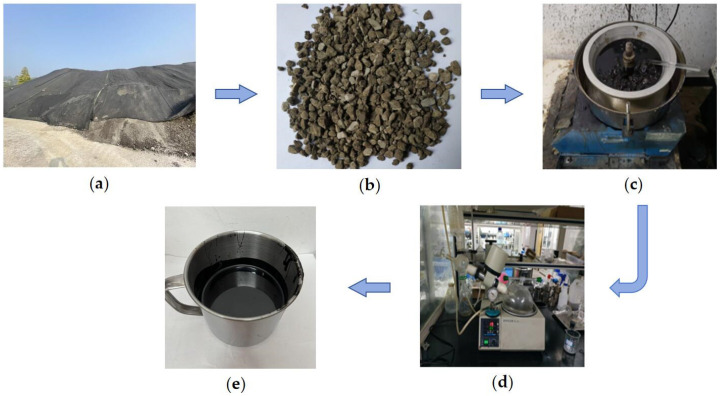
Process diagram of obtaining reclaimed asphalt.

Aged asphalt (AA) was obtained in large quantities through laboratory simulation to replace reclaimed asphalt (RA) in RAP. And the RAP was provided by Chongqing Zhongjiao Renewable Resources Development Co., Ltd. (Chongqing, China), as shown in Figure 2a,b. Firstly, asphalt leaching solution was prepared by dissolving RAP with trichloroethylene using the centrifugal separation method. Then, leachate was poured into a rotary evaporator to remove trichloroethylene and obtain RA, as shown in Figure 2c–e. Aged asphalt was produced based on Zhonghai 70# virgin asphalt (VA) by simulating the aging process in the laboratory [33]. Previous studies have shown that the accelerated aging of asphalt can be carried out using the rolling thin-film oven test (RTFOT) [34]. Therefore, virgin asphalt was heated using the RTFOT and its aging degree was evaluated every 30 min using the penetration test. It was found that the RTFOT of virgin asphalt at 163 °C for 180 min can simulate the properties of RA. To analyze the regeneration effect of warm-mix rejuvenators, AA and VA were used as control groups in this study.

Warm-mix recycled asphalt binders were prepared to evaluate the regeneration effect of the warm-mix rejuvenators on aged asphalt, accomplished by blending aged asphalt and BWR (or ZJ-WR) at 160 °C for 1 h. The weight ratios of BWR to aged asphalt were 3.5% to 11.5% at a 2% interval. The “3.5% BWR” in this study implies that the dosage of BWR in aged asphalt is 3.5% by mass. According to the data provided by the manufacturer, the dosage range of ZJ-WR is 6~8% by mass of aged asphalt. Hence, the dosage of ZJ-WR was selected as 7.5%, which was consistent with 7.5% BWR.

### 2.2. Experimental Methods

#### 2.2.1. Conventional Physical Tests

In order to investigate the regeneration effect of warm-mix rejuvenators on the conventional physical properties of aged asphalt, the penetration (25 °C), softening point, ductility (15 °C), and viscosity (135 °C) of virgin asphalt, aged asphalt, and warm-mix recycled asphalt were tested by ASTM D5, ASTM D36, ASTM D113, and ASTM D316. The shear rate for measuring the viscosity of various types of asphalt at 135 °C was 20 rpm/min.

#### 2.2.2. Temperature Sweep Test

The temperature sweep test of the dynamic shear rheometer (DSR) was used to measure the complex modulus (G*) and phase angle (*δ*) to characterize the viscoelasticity of asphalt binders at different high temperatures. And the rutting factor (G*/sinδ) was calculated, which reflected the deformation resistance of asphalt binders when the temperature increased. The test temperature ranged from 46 to ~82 °C in 6 °C increments with a frequency of 10 rad/s and strain level of 12%. The diameter and gap of parallel plate geometry were 25 mm and 1 mm, respectively.

#### 2.2.3. Multiple Stress Creep Recovery (MSCR) Test

The MSCR test was used to evaluate the high-temperature performance of asphalt binders by measuring the accumulation of non-recoverable creep deformation. The test temperature was 64 °C, and the shear stresses were 0.1 kPa and 3.2 kPa with 10 cycles of each stress level, respectively. Each cycle consists of 1 s of shear creep under load and 9 s of recovery after load removal. The percent recovery (*R*) and non-recoverable creep compliance (*J_nr_*) calculated with Equations (1) and (2) were used to evaluate the high-temperature performance of asphalt binders.

(1) $$R=\frac{\gamma_{p}-\gamma_{nr}}{\gamma_{p}-\gamma_{0}}\times 100\%$$

(2) $$J_{nr}=\frac{\gamma_{nr}-\gamma_{0}}{\tau }\times 100\%$$

where *γ*_0_ is the initial strain of each creep recovery cycle; *γ_p_* and *γ_nr_* are the peak strain and non-recoverable strain of each creep recovery cycle, respectively; and τ is the shear stress, kPa.

#### 2.2.4. Force–Ductility Test (FDT)

**Figure 3:**
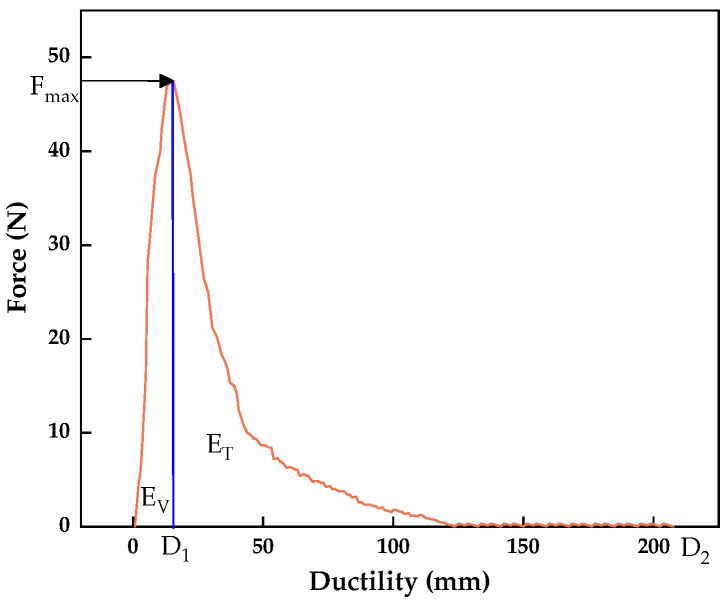
Schematic diagram of force–ductility.

The low-temperature performance of asphalt binders was evaluated at 10 °C using the force–ductility test with an “8” shaped mold. The tensile rate was 50 mm/min and the FDT was terminated when the tensile force reached 0/N. The tensile compliance (*f*), yield strain energy (*E_V_*), and toughness ratio (*R_T/V_*) obtained by the force–ductility curve were selected to evaluate the low-temperature rheological properties, as shown in Figure 3 and Equations (3)–(5) [35].

(3) $$f=D_{1}/F_{\max}$$

(4) $$E_{V}=\int_{0}^{D_{1}}F(x)dx$$

(5) $$R_{V/T}=E_{V}/E_{T}$$

where *F_max_* is the maximum tensile force, N; *D*_1_ is the displacement corresponding to *F_max_*, mm; *F*(*x*) is the function of the force–ductility curve; *E_V_* is the work done by external force during the elongational process of asphalt binders from the beginning of deformation to the occurrence of yield, N·mm; and *E_T_* is the work of external force in the elongational process of asphalt binders from yielding to breaking, N·mm. 

#### 2.2.5. Fourier Transform Infrared Spectroscopy (FTIR) Test 

The change of functional groups of aged asphalt before and after adding warm-mix rejuvenators was analyzed using Fourier transform infrared spectroscopy (FTIR). The spectra ranged from 4000 cm^−1^ to 400 cm^−1^ at a resolution of 4 cm^−1^, and the test temperature was 25 °C. For further quantitative analysis of the effect of warm-mix rejuvenators on the microscopic characteristics of aged asphalt, the areas of sulfoxide (*A_S=O_*), carbonyl (*A_C=O_*), and alkyl (*A_C-H_*) were obtained through integral calculation of the characteristic peak area of the corresponding functional groups. *A_S=O_* is the peak area from 966 cm^−1^ to 1062 cm^−1^ centered around 1030 cm^−1^; *A_C=O_* is the peak area from 1676 cm^−1^ to 1733 cm^−1^ centered around 1700 cm^−1^; and *A_C-H_* is the sum of the peak area from 565 cm^−1^ to 840 cm^−1^ centered around 694 cm^−1^, from 848 cm^−1^ to 906 cm^−1^ centered around 862 cm^−1^, from 1336 cm^−1^ to 1430 cm^−1^ centered around 1372 cm^−1^, from 1430 cm^−1^ to 1494 cm^−1^ centered around 1463 cm^−1^, from 2820 cm^−1^ to 2881 cm^−1^ centered around 2858 cm^−1^, and from 2881 cm^−1^ to 2945 cm^−1^ centered around 2918 cm^−1^.

#### 2.2.6. Atomic Force Microscope (AFM) Test 

The AFM test requires a smooth sample surface. In this study, the asphalt samples were prepared by the thermal coating method [36]. Firstly, about 0.1 g of hot liquid asphalt was poured onto a glass slide with a cover glass on, and it was placed in an oven at 120 °C for 30 s to make the asphalt flow naturally to the entire cover glass, and then the sample was taken out horizontally to remove the cover glass. The AFM test was performed when the samples were cooled to room temperature.

The micro-morphology of asphalt binders was characterized by the tapping mode of a Brook Dimension Icon AFM. The scanning area of 30 μm × 30 μm (512 pixels × 512 pixels) was scanned at a rate of 1.5 Hz. The silicon probe used in the AFM test was 3.7 um in height, 160 um in length, and 40 um in width, and the elastic coefficient was 26 N/m. Based on the AFM results, the average roughness (*S_a_*), interfacial area ratio (*S_dr_*), and surface material volume (*S_V_*) automatically obtained using Nanoscope analysis software 1.7 were selected to characterize the 3D micro-morphology of asphalt binders. These roughness parameters are defined as follows:

(6) $$S_{a}=\frac{1}{N}\sum_{i}^{N}\left|Z_{i}\right|$$

(7) $$S_{dr}=\frac{1}{A}\left\{\iint_{A}\left[\sqrt{\left[1+{\left(\frac{\partial z(x,y)}{\partial x}\right)}^{2}+{\left(\frac{\partial z(x,y)}{\partial y}\right)}^{2}\right]}-1\right]dxdy\right\}$$

(8) $$S_{V}=V_{P}+V_{V}$$

where *Z_i_* is the height of a point over the entire morphology, nm; *N* is the number of peaks and valleys of the 3D micro-morphology; *A* is the surface area of the 3D micro-morphology, um^2^; *V_P_* is the volume of peaks above the zero plane, nm^3^; and *V_V_* is the volume of valleys below the zero plane, nm^3^.

## 3. Results

### 3.1. Conventional Physical Properties

Table 2 shows the conventional physical properties of reclaimed asphalt, aged asphalt, virgin asphalt, and warm-mix recycled asphalt, including penetration, softening point, ductility, and viscosity. It can be seen that the penetration, ductility, softening point, and viscosity of aged asphalt are close to those of reclaimed asphalt, and aged asphalt can be used instead of reclaimed asphalt for testing. Compared with virgin asphalt, the penetration and ductility of aged asphalt decreased, while the softening point and viscosity increased. With the addition of BWR into aged asphalt, the penetration and ductility gradually increased, while the softening point and viscosity decreased. This indicates that the self-developed agent (BWR) can successfully regenerate the conventional physical properties of aged asphalt. When the content of BWR reached 7.5%, the performance level of warm-mix recycled asphalt was equivalent to that of virgin asphalt. With a further increase in BWR content, the penetration and ductility of aged asphalt were higher than those of virgin asphalt, while the softening point and viscosity were lower. The regeneration effect of BWR on the conventional physical properties of aged asphalt was better than that of ZJ-WR under the same dosage, which may be attributed to the lower viscosity of BWR than ZJ-WR.

### 3.2. Rheological Properties

#### 3.2.1. Temperature Sweep Test Results

**Figure 4:**
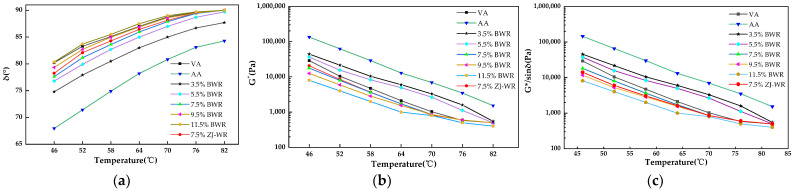
Temperature sweep test results of DSR: (**a**) phase angle; (**b**) complex modulus; (**c**) rutting factor.

Figure 4a,b show the phase angle (*δ*) and complex modulus (G*) obtained by the temperature sweep test, respectively. It can be seen that the phase angle of all asphalt binders gradually increased and the complex modulus decreased with the increase in temperature. Compared with virgin asphalt, the phase angle of aged asphalt decreased and the complex modulus increased at the same temperature. After incorporation of BWR, the phase angle of aged asphalt increased and the complex modulus decreased, which indicates that BWR can soften aged asphalt and improve its viscoelastic ratio. When the content of BWR reached 7.5%, the complex modulus of aged asphalt could be restored to the level of virgin asphalt, while the phase angle of aged asphalt could be restored to the level of virgin asphalt when the content of BWR reached 11.5%. The regeneration effects of BWR and ZJ-WR on the phase angle and complex modulus of aged asphalt were similar.

It can be seen from Figure 4c that the rutting factor (G*/sinδ) of all asphalt binders decreased with the increase in temperature. The rutting factor of asphalt increased significantly after aging, which indicates that its high-temperature rutting resistance was better than that of virgin asphalt. The incorporation of BWR reduced the rutting factor of aged asphalt when the BWR content exceeded 5.5%, and the rutting factor of aged asphalt could be restored to the level of virgin asphalt, which can be attributed to the regeneration effect of BWR on aged asphalt. However, excessive BWR content would further reduce the rutting factor of aged asphalt and damage its high-temperature performance. Compared with ZJ-WR, the regeneration effect of BWR on the rutting factor was closer to that of virgin asphalt.

#### 3.2.2. MSCR Test Results

**Figure 5:**
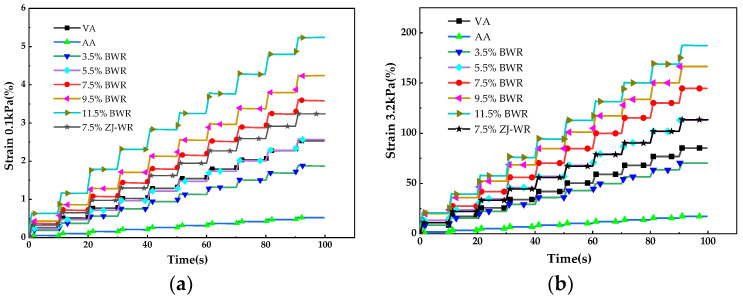
Creep recovery curve of MSCR test: (**a**) 0.1 kPa; (**b**) 3.2 kPa.

Figure 5a,b show the creep recovery curves of asphalt binders under shear stress values of 0.1 kPa and 3.2 kPa for 10 cycles, respectively. Compared with virgin asphalt, the cumulative strain of aged asphalt under different stresses was lower, but the cumulative strain increased after adding BWR. With the increase in BWR content, the strain of aged asphalt increased in each cycle, indicating that the stress sensitivity of aged asphalt was improved by adding warm-mix rejuvenators. The strain level of various asphalt binders was higher at 3.2 kPa, which suggested that the resistance to deformation of asphalt binders was reduced at a higher stress level. When the BWR content reached 5.5%, the strain of aged asphalt under different stresses could be restored to the level of virgin asphalt.

**Figure 6:**
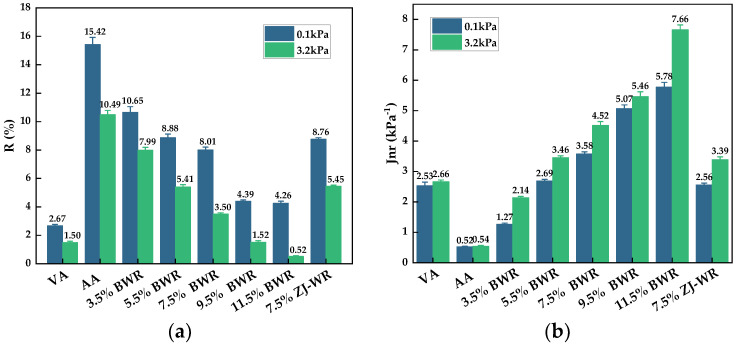
High-temperature performance parameters of MSCR: (**a**) percent recovery (*R*); (**b**) non-recoverable creep compliance (*Jnr*).

The effects of aging and regeneration on the elastic recovery and creep compliance of asphalt binders were further studied using the MSCR test. Figure 6a,b present the percent recovery (*R*) and non-recoverable creep compliance (*J_nr_*) under shear stress values of 0.1 kPa and 3.2 kPa, respectively. It can be seen from Figure 6 that compared with low stress levels (0.1 kPa), the *R* value of asphalt binders decreased at high stress levels (3.2 kPa), while the *J_nr_* value increased, indicating that the high-temperature deformation resistance of various asphalt decreased. In comparison with virgin asphalt, the *R* value of aged asphalt increased and the *J_nr_* value decreased, which contributed to stronger elasticity due to the increase in volatility of light components in asphalt, and the resistance to deformation was enhanced after aging. With the addition of BWR, the *R* value of aged asphalt decreased and the *J_nr_* value increased, indicating that the addition of warm-mix rejuvenators supplemented light components in aged asphalt and improved its viscoelastic properties. When the BWR content reached 7.5%, the *J_nr_* value could be restored to the level of virgin asphalt, while the *R* value could be restored by adding 9.5% BWR. And BWR had a better creep regeneration effect on aged asphalt than ZJ-WR.

#### 3.2.3. Force Ductility Test (FDT) Results

**Figure 7:**
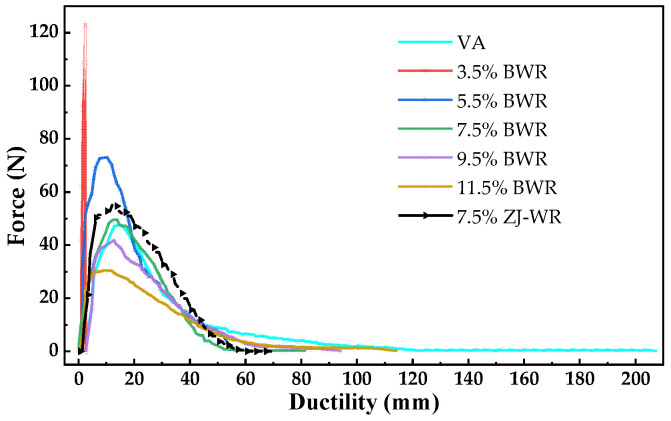
Force–ductility curves of various types of asphalt based on FDT.

It can be seen from Figure 7 that the low-temperature ductility of virgin asphalt was 207.6 mm. Due to the brittle fracture of aged asphalt at low temperatures (10 °C), there is no force–ductility curve of aged asphalt in Figure 7. When the content of BWR was low, the ductility recovery effect of aged asphalt was poor (the ductility of 3.5% BWR was only 2.8 mm). With the increase in BWR content, the low-temperature ductility of aged asphalt increased gradually. The low-temperature ductility of 11.5% BWR was 114.3 mm, which was only 55.1% of the ductility of virgin asphalt, indicating that the regeneration effect of warm-mix rejuvenators on the low-temperature ductility of aged asphalt was limited. The low-temperature ductility of 7.5% BWR was 81.5 mm, while that of 7.5% ZJ-WR was 69.1 mm, indicating that BWR had a better regeneration effect of ductility on aged asphalt than ZJ-WR.

According to Formulas (3)–(5), the low-temperature performance indexes of various types of asphalt calculated are shown in Table 3. The tensile compliance (*f*) of virgin asphalt was 0.33 mm·N^−1^, the yield strain energy (*E_V_*) was 444.66 N·mm, and the toughness ratio (*R_T/V_*) was 2.38. With the increase in BWR content, the *f* value and *R_T/V_* value of aged asphalt increased, while the *E_V_* value increased first and then decreased. The low-temperature deformation performance of asphalt binders was characterized by two aspects of force and displacement by the *f* value. The larger the *f* value, the stronger the low-temperature deformation ability of asphalt. The variation in asphalt before and after yield is presented using the *R_T/V_* value from the perspective of energy. The larger the *R_T/V_* value, the better the low-temperature stress-release ability of asphalt. Compared with the *f* value and *R_T/V_* value, the *E_V_* value did not fully reflect the low-temperature performance of aged asphalt, because it only characterized the stress accumulation degree of asphalt before yield [35].

When the content of BWR exceeded 7.5% and 9.5%, the *R_T/V_* values and *f* value of aged asphalt could be restored to the level of virgin asphalt, respectively. This implies that there may be some differences in the regeneration effect in evaluating the low-temperature performance of aged asphalt in terms of deformation and energy. And the regeneration effect of BWR and ZJ-WR on the low-temperature performance of aged asphalt was similar.

### 3.3. Micro-Morphology Analysis Based on AFM

#### 3.3.1. Two-Dimensional (2D) Bee-like Structures Analysis

**Figure 8:**
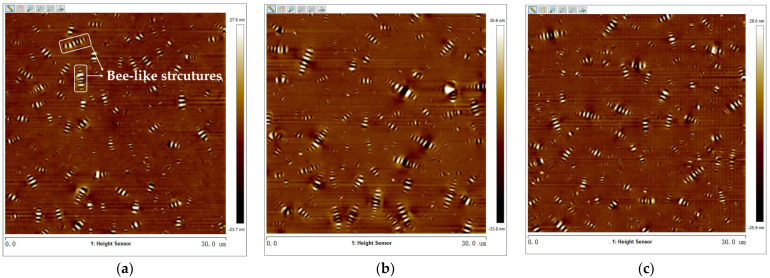

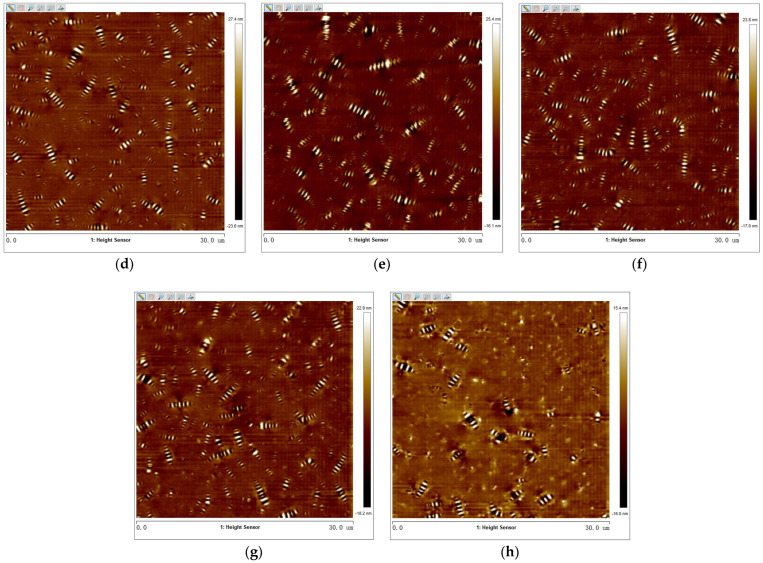
Two-dimensional micro-morphology of various asphalt binders: (**a**) virgin asphalt; (**b**) aged asphalt; (**c**) 3.5% BWR; (**d**) 5.5% BWR; (**e**) 7.5% BWR; (**f**) 9.5% BWR; (**g**) 11.5% BWR; (**h**) 7.5% ZJ−WR.

**Figure 9:**
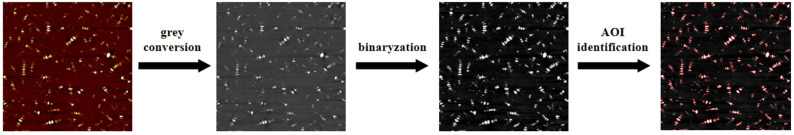
Image processing flowchart of bee-like structures extraction.

The surface micro-morphology of virgin asphalt, aged asphalt, and warm-mix recycled asphalt was obtained using the tapping mode of the AFM test. The 2D micro-morphology of various types of asphalt after processing by Nanoscope analysis software 1.7 is shown in Figure 8. It can be seen that there were obvious bee-like structures in asphalt. Previous studies have shown that bee-like structures were closely related to asphaltenes or wax crystals in asphalt [36]. The regeneration effect of warm-mix rejuvenators on aged asphalt can be evaluated on the micro-level by quantitatively analyzing the area and quantity of bee-like structures of aged asphalt and warm-mix recycled asphalt. The maximum area (S_max_), average area (S_mean_), total area (S_total_), and number (N) of bee-like structures in Figure 8 were extracted and counted using digital image analysis software (Image-Pro Plus 6.0). The image processing flowchart and statistical results are shown in Figure 9 and Table 4, respectively.

It can be determined from Table 4 that the *S_max_*, *S_mean_*, *S_total_*, and *N* values of bee-like structures of aged asphalt increased by 205%, 13.1%, 31%, and 15.2% compared with virgin asphalt, respectively. These results can be explained by the increase in and aggregation of asphaltenes which were transformed from aromatics and resins by aging action, observed as an increase in the area and number of bee-like structures in 2D morphology [37]. With the increase in BWR content, the *S_max_*, *S_mean_*, and *S_total_* values of bee-like structures of aged asphalt showed a trend of increasing first and then decreasing, and the N value showed a trend of decreasing first and then increasing, indicating that BWR may affect the formation and disaggregation of bee-like structures. In addition, the area parameters and number of bee-like structures of 7.5% ZJ-WR were largest, which was mainly caused by the image processing method. Therefore, in order to more accurately evaluate the regeneration effect of warm-mix rejuvenators on the micro-morphology of aged asphalt, the 3D surface micro-morphology should be further analyzed.

#### 3.3.2. Three-Dimensional (3D) Micro-Morphology Analysis

**Figure 10:**
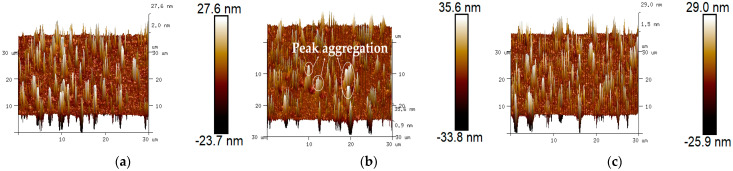

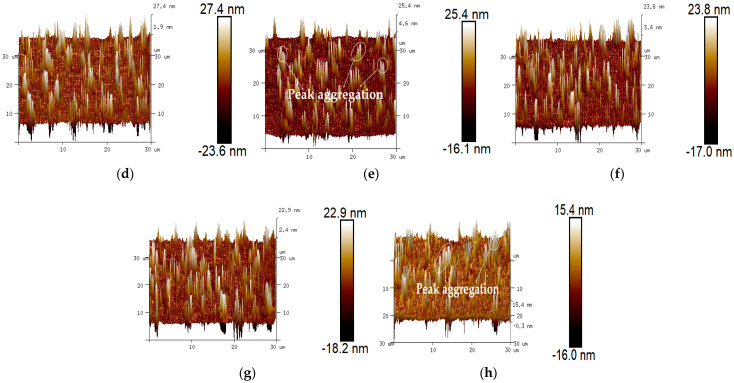
Three-dimensional micro-morphology of various asphalt binders: (**a**) virgin asphalt; (**b**) aged asphalt; (**c**) 3.5% BWR; (**d**) 5.5% BWR; (**e**) 7.5% BWR; (**f**) 9.5% BWR; (**g**) 11.5% BWR; (**h**) 7.5% ZJ−WR.

The 3D micro-morphology of virgin asphalt, aged asphalt, and warm-mix recycled asphalt can be visually seen in Figure 10, where it can be found that the 2D bee-like structures showed a peak–valley morphology at different heights in 3D view [38]. Compared with virgin asphalt, there is an obvious peak aggregation phenomenon of aged asphalt shown in Figure 10b, which can be attributed to the display of asphaltene aggregation in 3D space after aging [13]. The peak aggregation phenomenon of aged asphalt gradually decreased after adding BWR, but it did not seem to have a clear correlation with BWR content (there was still peak aggregation phenomena for 7.5% BWR). To quantitatively analyze the regeneration effect of warm-mix rejuvenators on the 3D micro-morphology of aged asphalt, roughness parameters such as average roughness (*S_a_*), interfacial area ratio (*S_dr_*), and surface material volume (*S_V_*) were used to characterize the variation in height, surface area, and peak–valley volume of aged asphalt before and after regeneration, respectively.

It can be seen from Table 5 that the *S_a_*, *S_dr_* and *S_V_* values of aged asphalt increased by 53.6%, 42.1%, and 63.9% after aging, respectively. This shows that the effect of aging on asphalt is not only reflected in the increase in height but also in the increase in surface area and volume, which is a potential explanation to the variation in area of 2D bee-like structures after aging. With the addition of BWR, all roughness parameters of aged asphalt showed a consistent trend of increasing first and then decreasing, which was similar to the variation in *S_max_*, *S_mean_*, and *S_total_* values of 2D bee-like structures. The same trends indicate that in addition to the commonly used roughness parameter (*S_a_*), the interface area ratio (*S_dr_*) and peak–valley volume (*S_dr_*) can also effectively characterize the evolution of the 3D micro-morphology of aged asphalt after regenerating. Compared with other types of asphalts, the roughness parameters of 7.5%ZJ-WR were smallest, which was contrary to the statistical results of area parameters of 2D bee-like structures shown in Table 5. The reason for this opposite result is that the height, surface area, and volume of the 3D morphology were reduced by the deagglomeration effect of some light components in ZJ-WR, while other light components were adsorbed around the strong polar asphaltene, and then the area parameters of 2D bee-like structures increased after digital image processing. Therefore, it is more accurate to evaluate the regeneration effect of warm-mix rejuvenators on the micro-morphology of aged asphalt by roughness parameters. In comparison with ZJ-WR, the regeneration effect of BWR on the micro-morphology of aged asphalt was closer to the level of virgin asphalt at the same dosage.

### 3.4. FTIR Analysis Results

**Figure 11:**
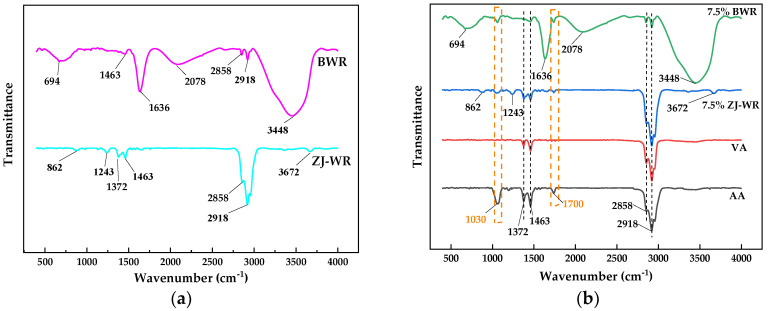
The FTIR spectrum: (**a**) BWR and ZJ−WR; (**b**) various asphalt binders.

FTIR was used to analyze the functional groups of warm-mix rejuvenators and various asphalt binders to reveal the regeneration mechanism of aged asphalt. The FTIR spectra of BWR are shown in Figure 11a. The absorption peaks at 2918 cm^−1^ and 2858 cm^−1^ were due to the asymmetric and symmetric stretching vibrations of C-H in alkanes, respectively. The strong absorption peak at 2078 cm^−1^ was caused by the stretching vibration of C≡C in alkynes. The strong absorption peak at 1636 cm^−1^ was caused by the stretching vibration of C=O in ketones. The absorption peak at 1463 cm^−1^ corresponded to the in-plane bending vibration of C-H in alkanes. And the strong absorption peak at 694 cm^−1^ corresponded to the out-of-plane bending vibration of C-H in alkynes. These characteristic peaks indicate that the BWR contained a high percentage of saturated hydrocarbons, unsaturated hydrocarbons, and aliphatic ketones, which were mainly from the chemical composition of waste plant oil, epoxidized soybean oil, and naphthenic rubber oil. The strong absorption peak at 3448 cm^−1^ caused by the stretching vibration of -OH indicated that waste plant oil and oleic acid diethanolamide contained free hydroxyl, which was evidence of the hydrogen bond exiting in BWR.

The FTIR spectra of ZJ-WR are shown in Figure 11b. The absorption peaks at 2918 cm^−1^, 2858cm^−1^, and 1463cm^−1^ are similar to those of BWR. The absorption peak at 1372 cm^−1^ was caused by the shear vibration of C-H in alkanes, and the absorption peak at 862 cm^−1^ was caused by the out-of-plane bending vibration of C-H on the benzene ring. These characteristic peaks represent a high proportion of saturated hydrocarbons, which can be attributed to the chemical structure of linseed oil. The weak absorption peaks at 1243 cm^−1^ and 3672 cm^−1^ were caused by the C-O-C stretching vibration of esters and the stretching vibration of -OH, respectively. These are due to the chemical structure of glycerol monostearate-containing ester groups and hydroxyl.

The spectra of virgin asphalt, aged asphalt, 7.5% BWR, and 7.5% ZJ-WR are shown in Figure 11b. Compared with virgin asphalt, obvious absorption peaks at 1700 cm^−1^ and 1030 cm^−1^ caused by the stretching vibration of C=O in carbonyl acid and S=O in sulfoxide can be found in the spectra of aged asphalt. According to previous studies [28,39,40], these two functional groups are commonly used to evaluate the aging of asphalt. Compared with aged asphalt, the intensities of absorption peaks of 7.5% BWR and 7.5% ZJ-WR at 1700 cm^−1^ and 1030 cm^−1^ are not obvious, which may be associated with the fact that warm-mix rejuvenators supplement numerous light components to aged asphalt. This indicates that aged asphalt was successfully regenerated by BWR and ZJ-WR. Combined with the microscopic characteristics analysis of BWR and ZJ-WR, no obvious characteristic peaks caused by chemical reactions were found, and there seemed to be only physical dilution in the regeneration process of aged asphalt.

For further quantitative analysis of the regeneration effect of warm-mix rejuvenators on microscopic functional groups, the areas of sulfoxide (*A_S=O_*), carbonyl (*A_C=O_*), and alkyl (*A_C-H_*) were calculated and are shown in Table 6. It has been confirmed that aged asphalt has larger values of *A_S=O_* and *A_C=O_* and a smaller value of *A_C-H_* compared with virgin asphalt [28]. As expected, the *A_S=O_* and *A_C=O_* values increased and the *A_C-H_* value decreased after aging. In addition, 7.5% BWR and 7.5% ZJ-WR showed lower *A_S=O_* values and higher *A_C-H_* values, which indicated that warm-mix rejuvenators have a good regeneration effect on the characteristic functional groups of aged asphalt. However, the *A_C=O_* value of 7.5% BWR was almost unchanged compared with aged asphalt, indicating that the *A_C=O_* value may not be reasonable for evaluating the regeneration effect of warm-mix rejuvenators containing bio-oil [40,41].

## 4. Conclusions

This study investigated the effect of a new bio-based warm-mix rejuvenator (BWR) on regenerating aged asphalt, in comparison to a commercial warm-mix rejuvenator (ZJ-WR). A series of laboratory tests were used to evaluate the performance and micro-morphology of virgin asphalt, aged asphalt, and warm-mix recycled asphalt. In addition, the regeneration mechanism of BWR and ZJ-WR was investigated. According to the analysis results, the conclusions are as follows:

(1) The incorporation of BWR into aged asphalt can improve the penetration and ductility values and reduce the softening point and viscosity values. When the content of BWR was 7.5%, the conventional physical properties of aged asphalt can be completely restored to the level of virgin asphalt.
(2) Aged asphalt showed better high-temperature rutting resistance with higher G*/sinδ and *R* values and a lower *J_nr_* value than virgin asphalt, while lower *f*, *E_V_*, and *R_T/V_* values indicate that the low-temperature performance of aged asphalt was poorer. The addition of BWR showed a great improvement in low-temperature performance of aged asphalt and a negative effect on high-temperature performance.
(3) The area parameters of 2D bee-like structures and the roughness parameters of the 3D micro-morphology of asphalt were increased after aging, while these parameters could decrease to the level of virgin asphalt after adding BWR with an appropriate dosage, indicating that BWR has a good regeneration effect on the micro-morphology of aged asphalt. In comparison with 2D area parameters, 3D roughness parameters were more accurate in evaluating the variation in micro-morphology of aged asphalt after regeneration.
(4) There was no new functional groups that appeared after the regeneration of aged asphalt, indicating that BWR and ZJ-WR mainly regenerate aged asphalt through physical dilution. Aged asphalt showed lower *A_S=O_* and *A_C=O_* values and a higher *A_C-H_* value after regenerating by BWR and ZJ-WR, and *A_S=O_* and *A_C-H_* values were more appropriate for the restoration evaluation of aged asphalt than the *A_C=O_* value.
(5) Compared with ZJ-WR, BWR has a better regeneration effect on road performance and the micro-morphology of aged asphalt, and the properties of 7.5%BWR are closest to the level of virgin asphalt.

## Figures and Tables

**Table 1:** Properties of BWR and ZJ-WR.

Properties	BWR	ZJ-WR
Viscosity, mm^2^/s (60 °C)	462	589
Flash point, °C	245	270
Viscosity ratio, % (after TFOT, 163 °C)	1.89	1.26
Wt change, % (after TFOT, 163 °C)	2.36	2.7
Density, g/cm^3^ (25 °C)	0.89	0.92
State	Brown liquid	Yellow solid

**Table 2:** Conventional physical properties of various asphalt binders.

Type	Penetration (25 °C, 5 s, 100 g)/0.1 mm	Ductility (5 cm/min, 15 °C)/cm	Softening Point/°C	Viscosity (135 °C)/Pa·s
RA	28	6.5	68.8	2.487
AA	27.6	5.3	69.1	2.674
VA	74	112	48.6	0.375
3.5% BWR	47.5	23.2	55.9	1.029
5.5% BWR	62.3	69.6	53.4	0.558
7.5% BWR	75.1	102.7	49.7	0.389
9.5% BWR	103.7	119.2	47.4	0.301
11.5% BWR	128.4	128.6	44.9	0.273
7.5% ZJ-WR	77.1	98.7	50.4	0.476

**Table 3:** Low-temperature performance indexes of various types of asphalt for force–ductility curves.

Type	*F_max_*/N	*D*_1_/mm	*f*/mm·N^−1^	*E_V_*/N·mm	*R_T/V_*
VA	47	15.5	0.33	445	2.38
3.5% BWR	123	2.6	0.02	111	0.09
5.5% BWR	73	10.3	0.14	554	2.13
7.5% BWR	50	13.0	0.26	427	2.16
9.5% BWR	48	12.9	0.31	316	2.91
11.5% BWR	30	10.4	0.34	238	3.54
7.5% ZJ-WR	55	13.8	0.25	439	2.19

**Table 4:** Statistical results of parameters of bee-like structure.

Type	*S_max_*/um^2^	*S_mean_*/um^2^	*S_total_*/um^2^	*N*
VA	0.37	0.084	24.2	289
AA	1.13	0.095	31.7	333
3.5% BWR	0.53	0.075	28.3	380
5.5% BWR	0.78	0.083	30.4	369
7.5% BWR	0.86	0.109	32.2	297
9.5% BWR	0.73	0.087	30.6	351
11.5% BWR	0.67	0.085	31.0	364
7.5% ZJ-WR	2.68	0.167	71.6	429

**Table 5:** Statistical results of roughness parameters.

Type	*S_a_*/nm	*S_dr_*/nm^2^	*S_V_*/nm^3^
VA	1.94	4.51	0.119
AA	2.98	6.41	0.195
3.5% BWR	2.09	4.77	0.140
5.5% BWR	2.35	5.09	0.145
7.5% BWR	2.17	4.38	0.089
9.5% BWR	1.86	3.86	0.073
11.5% BWR	2.05	4.15	0.087
7.5% ZJ-WR	1.68	3.30	0.042

**Table 6:** Functional group areas of asphalt binders.

Type	*A_S=O_*	*A_C=O_*	*A_C-H_*
VA	0.05	0.03	6.13
AA	2.97	0.58	4.07
7.5% BWR	0.94	0.57	7.39
7.5% ZJ-WR	0.73	0.21	5.61

## Data Availability

No new data were created or analyzed in this study. Data sharing is not applicable to this article.
